# Complete Genome Sequences of Priestia megaterium Type and Clinical Strains Feature Complex Plasmid Arrays

**DOI:** 10.1128/MRA.00403-21

**Published:** 2021-07-08

**Authors:** Philip S. Shwed, J. Crosthwait, K. Weedmark, E. Hoover, F. Dussault

**Affiliations:** a Environmental and Radiation Health Sciences Directorate, HECSB, Health Canada, Ottawa, ON, Canada; b Food Directorate, Bureau of Microbial Hazards, Health Canada, Ottawa, ON, Canada; c Food Directorate, Bureau of Food Surveillance and Science Integration, Health Canada, Ottawa, ON, Canada; University of Rochester School of Medicine and Dentistry

## Abstract

Here, we report the high-quality complete genome sequences and plasmid arrays of Priestia megaterium ATCC 14581^T^ and of two clinical strains (2008724129 and 2008724142) isolated from human samples in the United States.

## ANNOUNCEMENT

Priestia megaterium (basonym: Bacillus megaterium) is a common environmental bacterium that was first documented by Anton De Bary in 1884 (1) and is significant in biotechnology for the production of enzymes, recombinant proteins, and vitamins and for bioremediation activities (2). *P. megaterium* has been isolated from soil and seawater (2), indoor air (3), hospital environments (4), human infections (5), and blood samples (6). The genome of *P. megaterium* typically contains substantial plasmid content, and the genes carried on these mobile genetic elements are believed to contribute to the survival of the bacterium in diverse habitats (2). In order to understand the genetic background of selected clinical strains, we sequenced the complete genomes of two isolates collected from Laboratory Response Network sentinel laboratories in Rhode Island (Centers for Disease Control and Prevention isolates 2008724129 and 2008724142 [6]) and the type strain *P. megaterium* ATCC 14581.

Clinical strains were preserved as secondary –80°C glycerol stocks. ATCC 14581 was obtained from the American Type Culture Collection (ATCC). All strains were streaked onto brain heart infusion (BHI) agar plates, and single colonies were grown in 2 ml of BHI medium at 30°C with shaking at 220 rpm for 24 h. DNA was extracted using the MasterPure Gram-positive DNA purification kit (Lucigen Corporation) and treated with RNase A (2 µl at 10 mg/ml, 30 min at 37°C) and purified using solid-phase reversible immobilization selection (7). Short-read whole-genome sequences were collected on the MiSeq platform (v3 chemistry) using the 2 × 300-bp paired-end read protocol and Nextera XT library prep kits according to the manufacturer’s instructions (Illumina, Inc.). The short-read genome sequence of *P. megaterium* ATCC 14581^T^ was previously reported (8). Long-read sequencing was carried out on the MinION platform, and strain libraries were generated with PCR barcoding kit SQK-PBK004 and sequenced using the SQK-LSK108 kit on a FLO-106 flow cell as per the manufacturer’s instructions (Oxford Nanopore Technologies).

The hybrid genome assemblies were performed using both Nanopore and Illumina reads using Unicycler v0.4.8 (9) in bold mode and rotated using default parameters. Illumina read quality was assessed using FastQC v0.11.8 (http://www.bioinformatics.babraham.ac.uk/projects/fastqc). Raw sequencing reads were filtered to remove Illumina adapters, 3′ Q scores of <20, and reads of <15 bp using BBDuk v38 in the BBTools software suite (https://sourceforge.net/projects/bbmap/). The numbers of pre- and postfiltered Illumina reads were 3,790,156 and 3,789,803 (ATCC 14581^T^), 2,244,503 and 2,244,466 (CDC 2008724129), and 2,101,794 and 2,101,768 (CDC 2008724142), respectively. Following Guppy base calling (Guppy GPU v3.3.3+fa743ab), the MinION data set quality was analyzed using Nanoplot v1.20.0 (10), and MinION adapters were removed using Porechop v0.2.4 (https://github.com/rrwick/Porechop) with default settings. The numbers of pre- and postfiltered MinION reads, respectively, were 247,934 and 246,571 (ATCC 14581^T^), 171,448 and 170,600 (CDC 2008724129), and 48,664 and 48,466 (CDC 2008724142). The *N*_50_ values were 8,122 bp (ATCC 14581^T^), 8,956 bp (CDC 2008724129), and 7,768 bp (CDC 2008724142).

The NCBI Prokaryotic Genome Annotation Pipeline v4.13 (11) was used for annotation. Genome similarity was determined by the reciprocal best hit average nucleotide identity (two-way ANI) using the type strain genome server (12). The BLASTn algorithm was used to validate poly-γ-d-glutamic acid (PDGA) capsule gene presence against B. anthracis plasmid pXO2 (GenBank accession number NC_012655.1).

The closed genomes of *P. megaterium* ATCC 14581^T^, CDC 2008724129, and CDC 2008724142 had 313-, 178-, and 174-fold coverages, respectively. At 5.4 Mbp, strain CDC 2008724142 is comparable in size to ATCC 14581^T^ and has a high genome similarity reflected by a two-way ANI value of 99.3%. Strain CDC 2008724129 also has a high genome similarity to ATCC 14581^T^ with a two-way ANI value of 95.4%, although it is 0.6 Mbp smaller.

All the genomes feature genes for poly-γ-glutamate synthase (*pgsB*, -*C*, and -*A*) that account for the previous observation of capsules, antigenically similar to the PDGA capsule of B. anthracis (6). The major discriminators between the three strains are the plasmid/megaplasmid arrays and associated gene contents that are more numerous in the clinical strains than in the type strain. The closed genomes of *P. megaterium* ATCC 14581^T^ and two clinical strains may provide insights into comparative genomic analyses and the adaptations that have taken place in medical environments.

### Data availability.

The complete genome sequences of *P. megaterium* ATCC 14581^T^, the clinical strains, and the plasmids have been deposited in GenBank under the accession numbers shown in Table 1 and under BioProject PRJNA658106. The raw sequence data were deposited in the SRA database as follows: *P. megaterium* ATCC 14581^T^, SRX9430688 and SRR12978882; isolate 2008724129, SRR12978825 and SRR12978880; and isolate 2008724142, SRR12978821, SRR12978823, and SRR12978881.

**TABLE 1:** Genome assembly metrics of *P. megaterium* type and clinical strains

Strain	Accession no.	Name/plasmid	Size (bp)	G+C content (%)	Topology	No. of coding sequences
ATCC 14581^T^	CP069288	Chromosome	5,344,063	38.1	Circular	5,377
	CP069289	pPmT-1	157,528	34.8	Circular	168
	CP069290	pPmT-2	143,745	34.3	Circular	151
	CP069291	pPmT-3	74,778	34.6	Circular	69
	CP069292	pPmT-4	11,064	35.1	Circular	10
	CP069293	pPmT-5	10,610	33.6	Circular	11
	CP069294	pPmT-6	3,766	36.0	Circular	3
	CP069295	pPmT-7	2,048	35.4	Circular	1
CDC 2008724129	CP069397	Chromosome	4,835,211	38.4	Circular	4,853
	CP069398	pPmC-129-1	188,115	34.9	Circular	187
	CP069399	pPmC-129-2	124,513	34.8	Circular	125
	CP069400	pPmC-129-3	115,923	39.0	Linear	113
	CP069401	pPmC-129-4	86,772	35.9	Circular	74
	CP069402	pPmC-129-5	61,040	36.6	Circular	49
	CP069403	pPmC-129-6	57,784	34.6	Circular	50
	CP069404	pPmC-129-7	35,433	38.1	Linear	36
	CP069405	pPmC-129-8	28,387	38.9	Linear	25
	CP069406	pPmC-129-9	12,225	34.4	Circular	13
	CP069407	pPmC-129-10	9,349	34.7	Circular	7
	CP069408	pPmC-129-11	8,381	34.2	Circular	7
	CP069409	pPmC-129-12	8,052	34.7	Circular	9
	CP069410	pPmC-129-13	7,421	36.9	Circular	8
	CP069411	pPmC-129-14	7,313	33.7	Circular	9
	CP069412	pPmC-129-15	7,312	37.5	Circular	7
	CP069413	pPmC-129-16	4,697	37.1	Circular	5
	CP069414	pPmC-129-17	4,359	35.3	Circular	5
	CP069415	pPmC-129-18	3,251	36.1	Circular	2
	CP069416	pPmC-129-19	1,702	36.3	Circular	1
	CP069417	pPmC-129-20	1,680	36.6	Linear	1
CDC 2008724142	CP069606	Chromosome	5,416,261	38.1	Circular	5,420
	CP069607	pPmC-142-1	180,233	35.3	Circular	193
	CP069608	pPmC-142-2	152,532	35.0	Circular	143
	CP069609	pPmC-142-3	76,096	35.9	Circular	64
	CP069610	pPmC-142-4	59,258	33.0	Circular	52
	CP069611	pPmC-142-5	51,966	33.3	Circular	52
	CP069612	pPmC-142-6	15,650	34.7	Circular	16
	CP069613	pPmC-142-7	9,026	36.4	Circular	9
	CP069614	pPmC-142-8	9,000	33.9	Circular	11
	CP069615	pPmC-142-9	8,954	34.7	Circular	8
	CP069616	pPmC-142-10	7,782	33.3	Linear	8
	CP069617	pPmC-142-11	7,717	35.0	Circular	8
	CP069618	pPmC-142-12	3,778	36.0	Circular	3
	CP069619	pPmC-142-13	1,702	36.4	Circular	1
